# Successful management with endovascular stent graft repair following gunshot wound to the subclavian artery: Case report and literature review

**DOI:** 10.1016/j.ijscr.2019.09.040

**Published:** 2019-09-28

**Authors:** Adel Elkbuli, Saamia Shaikh, Mark McKenney, Dessy Boneva

**Affiliations:** a Department of Surgery, Kendall Regional Medical Center, Miami, FL, United States; b Department of Surgery, University of South Florida, Tampa, FL, United States

**Keywords:** Subclavian artery, Axillary artery, Stent graft repair, Endovascular repair, Trauma outcomes

## Abstract

**Introduction:**

Penetrating injuries to the subclavian artery are usually the result of gunshot wounds or stab wounds. While subclavian artery injuries are relatively uncommon, vascular injuries due to penetrating trauma are frequently encountered at Trauma Centers. Despite advances in modern medicine, these injuries are associated with a high mortality and can lead to devastating morbidity.

**Presentation of case:**

We report a case of a 20-year-old male who presented after sustaining multiple gunshot wounds to his left upper and lower extremities. He underwent an emergent repair of a left axillo-subclavian artery injury via an endovascular approach using a covered self-expanding stent and was discharged after less than a week.

**Discussion:**

Historically, open surgical repair was considered the gold standard in the management of subclavian artery injury. However, rapid technological developments and advances in vascular surgery offer alternative management approaches in traumatic vascular surgery. In a select subset of trauma patients with penetrating vascular injuries, a minimally invasive endovascular approach may be an option. Endovascular repairs are associated with shorter operative times, less blood loss, lower complications and also a reduced mortality rate.

**Conclusion:**

Endovascular stent graft prostheses offer a minimally invasive treatment modality in the management of traumatic penetrating subclavian artery injuries.

## Highlights

• Penetrating injuries to the subclavian artery are usually the result of stab wounds or gunshot wounds.
• Endovascular stent graft repair of traumatic subclavian artery injuries is a feasible alternative to open surgical approach.
• Several studies have reported that endovascular approach is associated with a decreased morbidity and mortality compared to open approach.

## 1 Introduction

The overall incidence of subclavian and axillary artery injuries, which are most commonly due to penetrating injury, account for less than 9% of all vascular injuries [[1], [2], [3]]. In particular, in the United States the most common mechanism causing this type of vascular injury is a gunshot wound [4]. Subclavian artery injuries are associated with a high mortality and despite advances of modern medicine remain extremely lethal injuries [5]. Management is particularly challenging due to anatomic location with the proximal third of the subclavian artery located within the thoracic cavity, hindering exposure, and because of the numerous delicate neurovascular structures in the vicinity, increasing the risk of collateral damage during repair or associated injuries [6]. The subclavian artery is protected by the subclavius muscle, the clavicle, the first rib, and the deep cervical fascia, as well as the costocoracoid ligament and the condensed clavi-coraco-axillary fascia. In addition to the difficult exposure, open repairs are associated with longer operative times, increased length of hospital stay, and a higher incidence of post-operative complications [2,3]. Endovascular approaches offer several technical advantages over the traditional open surgical approach and have become increasingly common in the last decade. We report a case of a gunshot wound victim who sustained an injury to the left subclavian and axillary arteries who was promptly and successfully treated via a percutaneous endovascular approach with a covered self-expanding stent. This case has been reported in line with the SCARE criteria [7].

## 2 Presentation of case

**Fig. 1:**
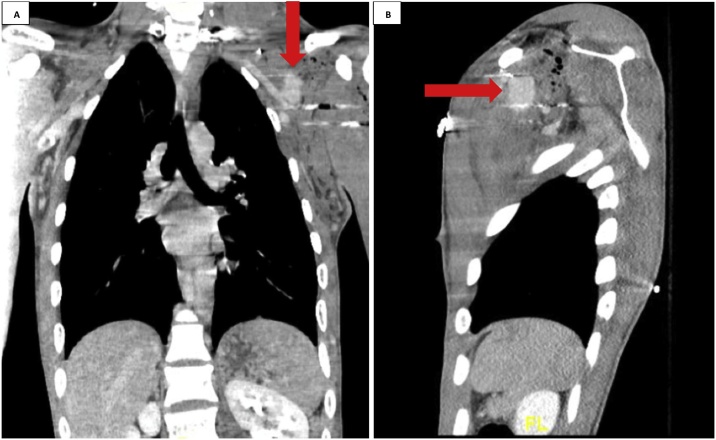
CT Angiography Chest. **A.** Coronal view: visualized above is a left subclavian/axillary artery injury with contrast extravasation at the site of injury along the course of the artery coursing out of the neck and in to the left shoulder. **B.** Sagittal view: a large amount of extravasation is seen in the left shoulder area along with an expanding hematoma. Subcutaneous emphysema is also visualized.

**Fig. 2:**
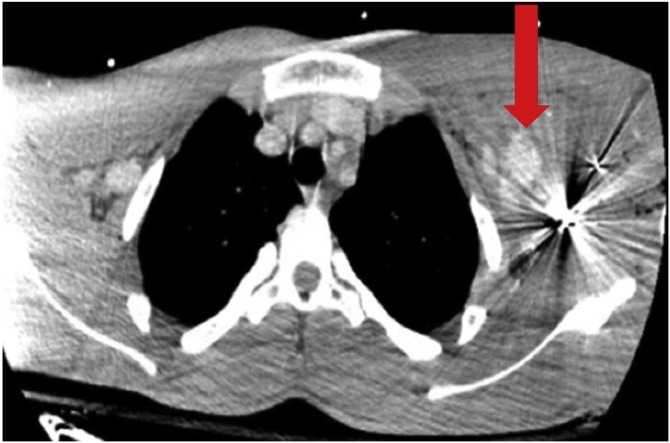
CT Angiography Chest. Gunshot wound to the left shoulder; the bullet visualized above creates artifact at the left shoulder thus obscuring visibility of the arterial injury. Nevertheless, extravasation of contrast material is evident. A large caliber bullet fragment is also noted overlying the left shoulder.

**Fig. 3:**
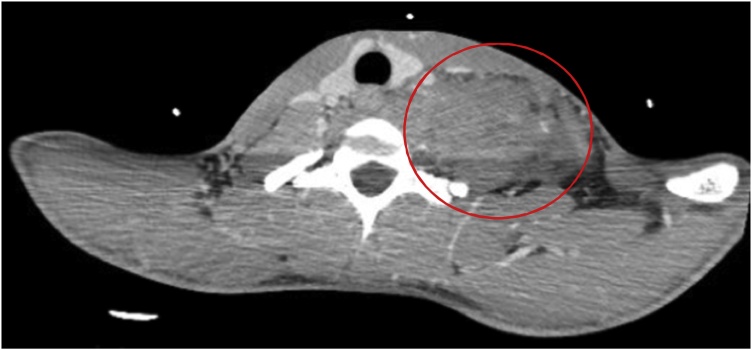
CT Angiography Chest. A large, rapidly expanding hematoma creating an asymmetrical enlargement with slight deviation of the trachea to the right – away from the hematoma. Subcutaneous emphysema is noted in this image as well.

The patient is a previously healthy 20-year-old male who presented to our trauma bay after sustaining multiple gunshot wounds of the left upper and lower extremities. On vascular examination, he had a diminished left radial pulse. Computed Tomography (CT) imaging revealed a left axillo-subclavian injury with an associated lung contusion and nondisplaced humerus fracture. CT Angiography revealed injury to the left subclavian artery, an active area of extravasation with a large pool of contrast in the axillary artery and an expanding hematoma (Fig. 1, Fig. 2, Fig. 3). Imaging did not reveal any vascular injury to the lower extremity.

**Fig. 4:**
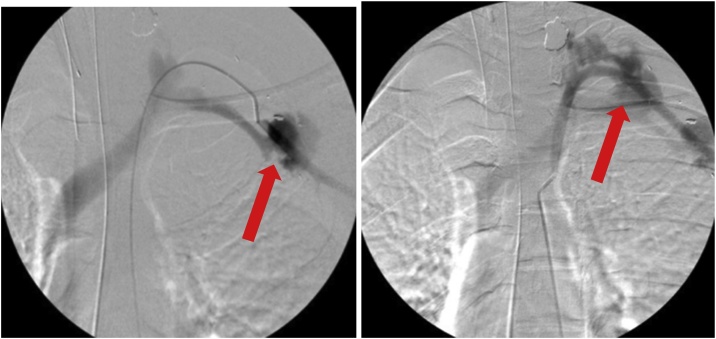
Diagnostic angiograms performed to better visualize injury. **A.** Extravasation of contrast material is seen close to the left clavicle on this angiogram view making this a difficult anatomical location for repair. **B.** Large amount of contrast extravasation indicating injury to the artery.

**Fig. 5:**
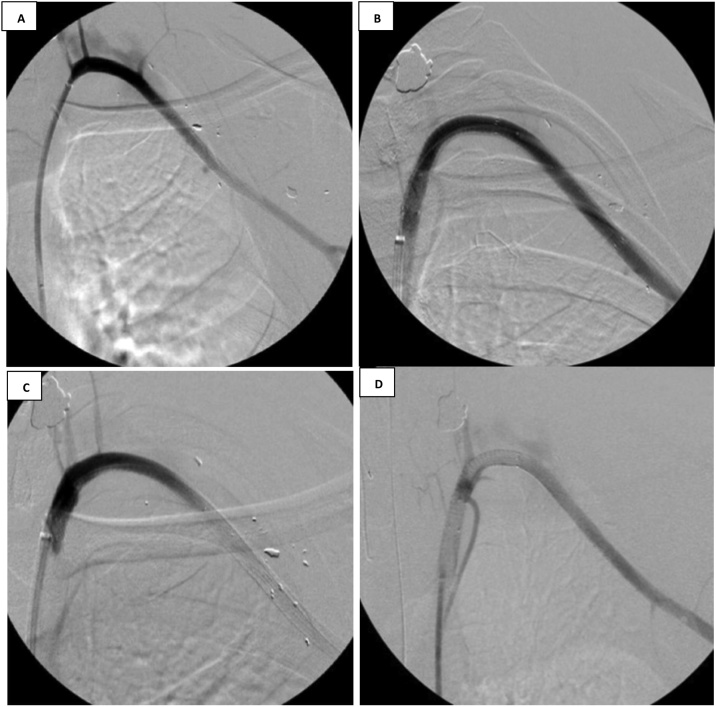
Completion angiograms demonstrated successful endograft exclusion. **A.** Angiogram of the left axillo-subclavian artery injury post Viabahn stent placement. **B.** Vascular flow without extravasation of contrast material immediately following stent placement. **C.** Arterial flow within the left subclavian artery is seen coming off of the aorta. **D.** The proximal Viabahn stent is well visualized here into the proximal left subclavian artery. Blood flow into the left subclavian and left axillary arteries are intact without extravasation.

The patient underwent emergent endovascular repair of this left axillo-subclavian injury. Intraoperatively, selective angiograms better characterized the injury (Fig. 4) and the decision was made to use a covered stent. Access was obtained via the common femoral artery. One 6 mm × 5 cm stent was deployed to the distal left subclavian artery and two stents, 7 mm × 5 cm and a 7 mm × 10 cm, were deployed to the proximal subclavian and axillary arteries. He received heparin throughout the procedure. Completion angiograms were performed to rule out an endoleak, and to confirm good distal runoff to the upper extremity (Fig. 5). The patient was taken to the ICU postoperatively. He had a palpable left brachial and radial pulse. Orthopedic surgery recommended non-operative management of the left open humerus fracture with 72 h of intravenous cefazolin. Antiplatelet therapy with aspirin 325 mg and venous thromboembolism prophylaxis with enoxaparin were initiated. He was transferred from the ICU to the surgical floor on POD#2 and was discharged to home on POD#5. On follow up the patient had a patent stent graft without endoleak.

## 3 Discussion

Subclavian artery injuries have traditionally been treated with open surgical intervention. Open repair of the subclavian artery is challenging for even the most experienced of surgeons due to the tunnel of protection it is afforded by the thoracic cavity [8,9]. Surgical repair of vascular injury in the trauma setting is also often complicated by associated venous injuries, soft tissue injury and hematoma formation, exacerbating the challenging nature of such a repair [8]. Open repairs have also been associated with mortality rates as high as 30% [3]. Thus, management of these injuries continue to evolve with endovascular approaches becoming more common. Given the rarity of subclavian injuries, large-scale studies are highly unlikely. The largest retrospective review was by Demetrides et al. in which 79 subclavian and axillary artery injuries were examined over a 4-year period [10]. The authors revealed arterial injuries in 75% of patients, venous injuries in 50% of patients, and an unfortunate 25% with both arterial and venous injuries.

A spectrum of injuries to the subclavian artery have been reported in the literature and include pseudoaneurysms, arteriovenous fistulas, and partial and complete transections. Avulsions, occlusions, and dissections have also been reported [2,8,11]. In a 13-year retrospective review, penetrating injuries more often resulted in proximal subclavian artery injuries when compared to middle and distal injuries [12]. Furthermore, penetrating injuries in this area are usually the result of gunshot wounds and, less likely, stab wounds [13,14]. In a 10-year retrospective review, Lin and colleagues reported that the exact mechanism of injury was associated with outcome differences with stab wounds resulting in an 80% survival rate and gunshot wounds resulting in a 63% survival rate [13].

Associated injuries reported in the literature include vertebral artery injuries, carotid artery injuries, brachial plexus injuries, subclavian vein injuries, injuries to the aerodigestive tract, and injuries to the sympathetic chain and spinal cord [3,13,15]. In a case series by Cohen et al., all of the patients had an associated lung injury [3]. Consistent with the literature, our patient had an associated lung contusion. He also had an associated open proximal humerus fracture. Interestingly, in a retrospective analysis of the National Trauma Data Bank, Grigorian and colleagues reported an open proximal humerus fracture was the strongest risk factor for limb loss [15].

Endovascular approaches for penetrating arterial trauma, which are not limited to only subclavian artery injuries, include embolization and stent graft repair. Stent graft repair is often the preferred approach as it preserves blood flow [14]. The use of many different types of stents in the treatment of axillo-subclavian artery injuries have been reported. We used a covered self-expanding stent (GORE Viabahn Endoprosthesis® W.L. Gore & Associates, Flagstaff, AZ) that contains an expanded polytetrafluoroethylene layer to reduce postoperative complication rates [6,13,16]. The Food and Drug Administration (FDA) approved the use of this stent in 2005 and since then it has been used for a variety of injuries including occlusive arterial injury, arteriovenous fistulas, and pseudoaneurysms [17]. There are a number of factors which ultimately guide the type of stent to utilize including surgeon preference, availability, and the characteristics and location of the lesion. For injuries resulting from stab wounds and gunshot wounds, self-expanding covered stents are typically desired due to their flexibility and ability to conform to multiple diameters in a single artery [14]. This is especially useful for vessels which are difficult to fully visualize such as the subclavian artery. For the same reason, the Viabahn stent is a reasonable option as it can be oversized by 1 mm. The stent must be positioned with 1 cm of normal artery covered on both ends of the area of damage [13].

However, stent-related complications include stent fracture, stent thrombosis, and migration [17]. In addition, due to its relatively recent introduction into the clinical market, the lack of long-term data on outcomes continues to be of concern [17]. Another downside of endovascular approaches is that the damaged vessel itself cannot be directly visualized and also injury to adjacent structures cannot be directly seen as would be with an open surgical repair [18]. Further, the proximity of the vertebral artery to the subclavian artery predisposes it to branch vessel coverage which can potentially lead to a cerebrovascular incident [8]. Failure of endovascular repair is typically the result of an inability to pass the guidewire across the length of the lesion [3]. Notwithstanding, endovascular approaches offer several advantages over open approaches such as remote access, shorter operative time, less blood loss, lower incidence of sepsis, and a reduced risk of injury to surrounding structures [2,3,8,14,[17], [18], [19], [20], [21]]. Additionally, quicker recovery time and a decreased length of hospital stay are associated with endovascular approaches [22]. In a comparison of outcomes after endovascular repair and open operative repair, Branco et al. reported a statistically significant decrease in mortality (5.6% versus 27.8%, respectively; *P* = 0.04). The authors also reported a lower complication rate with the endovascular approach [5].

Hemodynamic instability was previously considered to be a contraindication to endovascular repair, but this is now controversial with several authors supporting endovascular repair in hemodynamically unstable patients [2,3]. Other contraindications to endovascular repair include complete transection of the subclavian artery and long segmental injuries [2].

Our case report is notable in that we describe a case of a patient with an actively extravasating subclavian artery injury and expanding hematoma which was successfully treated using emergent endovascular repair.

## 4 Conclusion

Endovascular intervention with stent graft repair is an alternative approach to open surgical repair in the management of traumatic penetrating axillo-subclavian artery injuries in select patients. Endovascular approaches are associated with a decreased blood loss, shorter operative times, reduced risk of injury to surrounding structures, lower complication rates, and decreased mortality.

## Sources of funding

None.

## Ethical approval

This report was conducted in compliance with ethical standards. Informed written consent has been obtained and all identifying information is omitted.

## Consent

Informed written consent has been obtained and all identifying information is omitted.

## Author contribution

Adel Elkbuli, Saamia Shaikh, Dessy Boneva, Mark McKenney – Conception of study, acquisition of data, analysis and interpretation of data.

Saamia Shaikh, Adel Elkbuli, Dessy Boneva – Drafting the article.

Dessy Boneva, Mark McKenney – Management of case.

Adel Elkbuli, Saamia Shaikh, Dessy Boneva, Mark McKenney – Critical revision of article and final approval of the version to be submitted.

## Registration of research studies

This is a case report study.

## Guarantor

Dessy Boneva.

Mark McKenney.

## Provenance and peer review

Not commissioned, externally peer-reviewed.

## Declaration of Competing Interest

None.
